# Therapeutic potential of traditional Chinese medicine in pancreatic fibrosis: mechanisms of TCM formulas and active ingredients

**DOI:** 10.3389/fphar.2025.1609569

**Published:** 2025-10-03

**Authors:** Kexin Gan, Yonghong Hu, Wei Liu, Jiewen Shi, Sen Yang, Jinxin Huang, Xuan Wang, Fu Li

**Affiliations:** ^1^ Department of Hepatobiliary Pancreatic Surgery, Shuguang Hospital Affiliated to Shanghai University of Traditional Chinese Medicine, Shanghai, China; ^2^ Department of Integrated Traditional Chinese and Western Medicine Surgery, Shuguang Hospital Affiliated to Shanghai University of Traditional Chinese Medicine, Shanghai, China; ^3^ Key Laboratory of Liver and Kidney Diseases (Ministry of Education), Department of Pharmacy, Institude of Liver Diseases, The NATCM Third Grade Laboratory of Traditional Chinese Medicine Preparations, Shanghai Key Laboratory of Traditional Chinese Clinical Medicine, Shuguang Hospital Affiliated to Shanghai University of Traditional Chinese Medicine, Shanghai, China; ^4^ Department of Laboratory Medicine, Shuguang Hospital Affiliated to Shanghai University of Traditional Chinese Medicine, Shanghai, China

**Keywords:** traditional Chinese medicine, chronic pancreatitis, pancreatic fibrosis, signaling pathways, review

## Abstract

Pancreatic fibrosis (PF), the primary pathological hallmark of chronic pancreatitis (CP), is recognized as a pivotal driver of CP progression. Currently, no therapies are approved by the U.S. Food and Drug Administration (FDA) specifically for PF treatment, highlighting an urgent need for novel therapeutic strategies. Emerging evidence positions Traditional Chinese Medicine (TCM) as a promising multi-target approach against PF. This paper summarizes the pathogenesis of PF and provides a detailed review and comprehensive analysis of the mechanisms underlying Chinese herbal formulas and active ingredients investigated for PF prevention and treatment in existing experimental studies. Numerous studies indicate that TCM combats PF by inhibiting pancreatic stellate cells (PSCs) activation, regulating extracellular matrix (ECM) breakdown, suppressing macrophage infiltration and polarization, and inhibiting pancreatic acinar cell apoptosis. Current basic research predominantly focuses on PSC activation and associated signaling pathways, particularly key pathways such as TGF-β/Smad, MAPK, NF-κB, and Hedgehog. This work thus offers novel insights and approaches for PF treatment and further research.

## Graphical Abstract

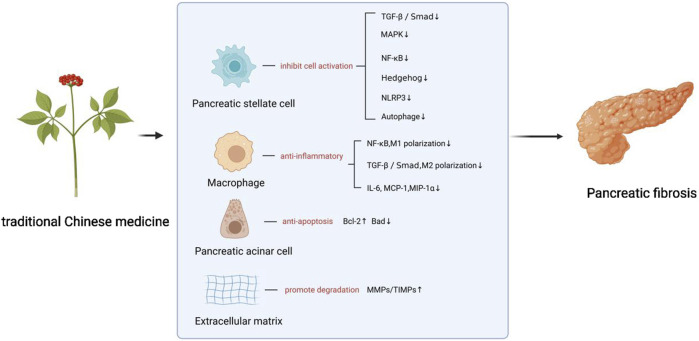

## 1 Introduction

Chronic pancreatitis (CP) is a multifactorial fibroinflammatory disorder characterized by recurrent pancreatic inflammation, culminating in extensive fibrotic tissue replacement. This process leads to chronic pain, exocrine and endocrine pancreatic insufficiency (Beyer et al., 2020). The annual incidence of CP is estimated at 5–14 per 100,000 individuals, while its prevalence ranges from 120 to 143 per 100,000 annually (Kleeff et al., 2017; Lévy et al., 2014). Pancreatic fibrosis (PF) is recognized as a central driver in CP pathogenesis. PF represents a chronic, progressive pancreatic pathology marked by extensive fibroblast proliferation and excessive accumulation of extracellular matrix (ECM) rich in connective tissue components (Ceyhan and Friess, 2015). This fibrotic cascade results in pancreatic parenchymal scarring, functional tissue loss, acinar cell atrophy, pancreatic ductal alterations, and inflammatory cell infiltration.

Current management of CP primarily involves symptomatic interventions, including analgesia and pancreatic enzyme supplementation, with suboptimal outcomes (Kichler and Jang, 2020). Endoscopic procedures, such as endoscopic retrograde cholangiopancreatography (ERCP) and surgical interventions, may provide sustained symptom relief. However, they neither restore pancreatic exocrine function nor halt the progression of PF (Dumonceau et al., 2019). Endoscopic approaches aim to reestablish pancreatic ductal drainage through techniques including sphincterotomy, stricture dilatation, stenting, stone extraction, and extracorporeal shock wave lithotripsy (ESWL). When endoscopic and conservative management fail, surgical intervention is indicated. Common procedures include longitudinal pancreaticojejunostomy, decompressive pancreatico-intestinal anastomoses, and various pancreatic head resections (Shimizu et al., 2022).

While traditional Chinese medicine (TCM) does not possess a direct pathological correlate to PF, its characteristic clinical manifestations, such as epigastric or abdominal pain, nausea, vomiting, anorexia, and diarrhea, align with TCM syndrome categories of “abdominal pain” and “diarrhea” Therapeutic strategies in TCM emphasize fortifying spleen qi, soothing liver qi stagnation, clearing heat, activating blood circulation, and resolving stasis. In contrast to Western medicine, which often focuses on specific symptoms or complications, TCM adopts a holistic approach centered on syndrome differentiation and pattern-based treatment. TCM offers potential advantages including clinical efficacy, reduced recurrence rates, and a favorable safety profile, making it a promising therapeutic avenue (Chen S et al., 2023; Huang et al., 2025). Recent pharmacological studies have increasingly demonstrated that TCM, which includes both TCM formulas and active ingredients, can inhibit the progression of PF (Liu C. et al., 2019). However, a comprehensive review that synthesizes these advances is still lacking. This study aims to review contemporary research progress on TCM interventions for PF and to provide novel insights and approaches for its treatment and further investigation.

## 2 Methods and literature search strategy

A systematic literature search was conducted to identify all relevant preclinical studies investigating TCM-derived compounds and formulas for PF and CP. The electronic databases PubMed, Web of Science Core Collection, and Google Scholar were searched from their inception until 1 February 2025. The search strategy combined keywords and Medical Subject Headings (MeSH) terms related to: (1) Intervention: (“traditional Chinese medicine” OR “Chinese herbal medicine” OR “natural product” AND (2) Disease: (“chronic pancreatitis” OR “pancreatic fibrosis”). Inclusion criteria were: (1) *in vitro* or *in vivo* studies; (2) studies investigating defined TCM compounds or chemically characterized extracts; (3) studies reporting outcomes related to PF mechanisms (e.g., PSC activation, ECM deposition, macrophage polarization). Exclusion criteria were: (1) reviews, editorials, or conference abstracts; (2) studies using undefined crude mixtures; (3) studies not published in English. Two investigators independently screened titles and abstracts, followed by a full-text review of potentially eligible articles. Any discrepancies were resolved through discussion with a third investigator.

In addition, literature should be excluded that includes “pan assay interfering compounds” (Magalhães et al., 2021). Readers may consult the original publications for precise statistical tests and exact p-value thresholds via the hyperlinked references.

All herbal medicines derived from plants have undergone taxonomic verification (http://mpns.kew.org/mpns-portal/) and include complete species names (including authoritative nomenclature and taxonomic classification). As the MPNS covers only plant-derived medicines, any medicines derived from fungal or animal are referred to by their standard names throughout this article.

## 3 Mechanisms of PF

Recent research indicates that the PF microenvironment comprises three principal cell types: pancreatic acinar cells, macrophages, and pancreatic stellate cells (PSCs) (Apte et al., 2011; Hu et al., 2020; Xue et al., 2015). Among these, PSC activation is pivotal to the initiation and progression of CP, with activated PSCs playing a major role in PF development (Masamune et al., 2009). Established risk factors for CP include smoking, heavy alcohol consumption, genetic disorders, pancreatic duct obstruction, recurrent acute pancreatitis, and autoimmune pancreatitis (Shimizu, 2008; Singh et al., 2019). Smoking and excessive alcohol intake induce acinar cell damage, triggering inflammatory cells (including macrophages) to secrete pro-inflammatory cytokines such as interleukin (IL)-1, IL-6, IL-8, tumor necrosis factor-α (TNF-α), transforming growth factor-β (TGF-β), and platelet-derived growth factor (PDGF) (Chang et al., 2023; Mews et al., 2002). These cytokines stimulate quiescent PSCs via paracrine signaling. Furthermore, damaged acinar cells directly release damage-associated molecular patterns (DAMPs) that facilitate PSC activation (An et al., 2023). Evidence also suggests that factors including smoking, heavy alcohol intake, and oxidative stress may directly activate quiescent PSCs (Chang et al., 2023; Fu et al., 2018). Upon activation, PSCs undergo a phenotypic transition characterized by α-smooth muscle actin (α-SMA) expression, enhanced proliferation and migration, and increased synthesis and secretion of ECM components (Apte et al., 1999; Bynigeri et al., 2017). Critically, activated PSCs secrete cytokines that perpetuate their activation through autocrine signaling. This cascade promotes excessive ECM deposition over degradation, ultimately driving PF pathogenesis (Jin et al., 2020). Collectively, these 3 cell types interact within the pathological microenvironment, synergistically promoting the progression of PF.

### 3.1 PSCs and PF

PSCs, the principal fibroblast population in the pancreas, reside in periacinar and interlobular regions. Like hepatic stellate cells, PSCs store retinol and fatty acid retinyl esters and express desmin (Bachem et al., 1998). These pluripotent cells constitute approximately 4%–7% of pancreatic parenchymal cells and are essential for maintaining connective tissue architecture (Xue et al., 2018).Under physiological conditions, PSCs maintain a quiescent state characterized by expression of nestin, vimentin, glial fibrillary acidic protein (GFAP), and desmin. Quiescent PSCs exhibit distinctive features, including large perinuclear lipid droplets, specific molecular markers (cytosolic bead proteins and lipophilic proteins), and limited capacities for proliferation, migration, and ECM synthesis (Nielsen et al., 2017). PSCs become activated by diverse inflammatory stimuli within the pancreatic microenvironment, primarily originating from macrophages and damaged acinar cells in CP. Upon activation, PSCs transition into a myofibroblast-like phenotype, characterized by the depletion of cytoplasmic lipid droplets and the concomitant upregulation of α-smooth muscle actin (α-SMA), various cytokines, and extracellular matrix (ECM) components, including collagen type I (Col-I), collagen type III (Col-III), hyaluronic acid (HA), and fibronectin (FN). Concurrently, their proliferative and migratory capacities increase. This activation drives excessive ECM deposition, resulting in interlobular and intralobular fibrosis.PSCs exhibit significant plasticity, capable of bidirectional transition between quiescent and activated states. Evidence suggests PF may be reversible in early stages (Apte et al., 2011; Kisseleva and Brenner, 2021). Given that PSCs activation is central to pathological fibrosis in CP, suppressing PSCs activation and promoting reversion to quiescence represent promising therapeutic strategies for PF management.

### 3.2 Pancreatic acinar cells and PF

Pancreatic acinar cells, responsible for secreting digestive enzymes, constitute a critical cell population within the pancreas and are intimately linked to CP pathogenesis (Saluja et al., 2019). Aberrant intra-acinar trypsinogen activation leading to acinar cell necrosis is considered a key initiating event in CP. These necrotic cells release DAMPs, promoting PSCs activation, macrophage infiltration, and polarization, thereby accelerating PF progression (Hoque et al., 2011; Kang et al., 2014). Consequently, injured acinar cells may directly stimulate PSCs or indirectly activate them through the secretion of profibrotic mediators (An et al., 2023). Furthermore, acinar cells can actively remodel the microenvironment to sustain persistent PSC activation (Liu J. et al., 2019). Supporting a direct role in fibrosis, acinar cells were identified as the primary collagen-producing cells in a caerulein-induced rat model of acute pancreatitis (Gong et al., 2013).

Pancreatic acinar cells exhibit significant regenerative capacity following injury. During inflammation, they undergo morphological changes, which involve a transition from tall, columnar cells to flattened configurations, and frequently encircle ductal structures. Phenotypically, this shift involves a conversion from predominantly amylase-positive acinar cells to cytokeratin 19 (CK19)-positive duct-like cells, defining acinar-to-ductal metaplasia (ADM), a hallmark feature of CP (Ma et al., 2022; Schlesinger et al., 2020). Macrophages have been implicated as key regulators driving this phenotypic alteration in acinar cells (Liou et al., 2013).

### 3.3 Macrophages and PF

The progression of PF is characterized by inflammatory cell infiltration, primarily consisting of macrophages along with lymphocytes and neutrophils, which collectively represent the dominant immune population invading the pancreas during CP (Michalski et al., 2007; Treiber et al., 2011). Pancreatic tissue injury triggers substantial macrophage recruitment and activation, initiating inflammatory cascades. When damaging stimuli persist and inflammation becomes chronic, sustained macrophage activation within the inflammatory milieu drives their polarization into distinct functional phenotypes in response to chemokines and cytokines. These polarized macrophage subsets exert divergent regulatory effects on PF progression.

Macrophages undergo functional polarization, which is a process of differentiation into distinct phenotypes dictated by microenvironmental cues and signaling molecules (Luo et al., 2024). Two principal polarization states are recognized: M1 (classically activated) and M2 (alternatively activated) macrophages. M1 macrophages are characterized by pro-inflammatory responses and anti-tumor activity, while M2 macrophages promote angiogenesis, anti-inflammatory mediator release, fibrosis, and tissue repair/wound healing (Gordon and Martinez, 2010). Emerging evidence demonstrates that activated PSCs stimulate macrophage polarization toward the pro-fibrotic M2 phenotype, thereby contributing to PF pathogenesis (Xue et al., 2015).

## 4 Molecular mechanisms and current research on TCM for PF

**FIGURE 1:**
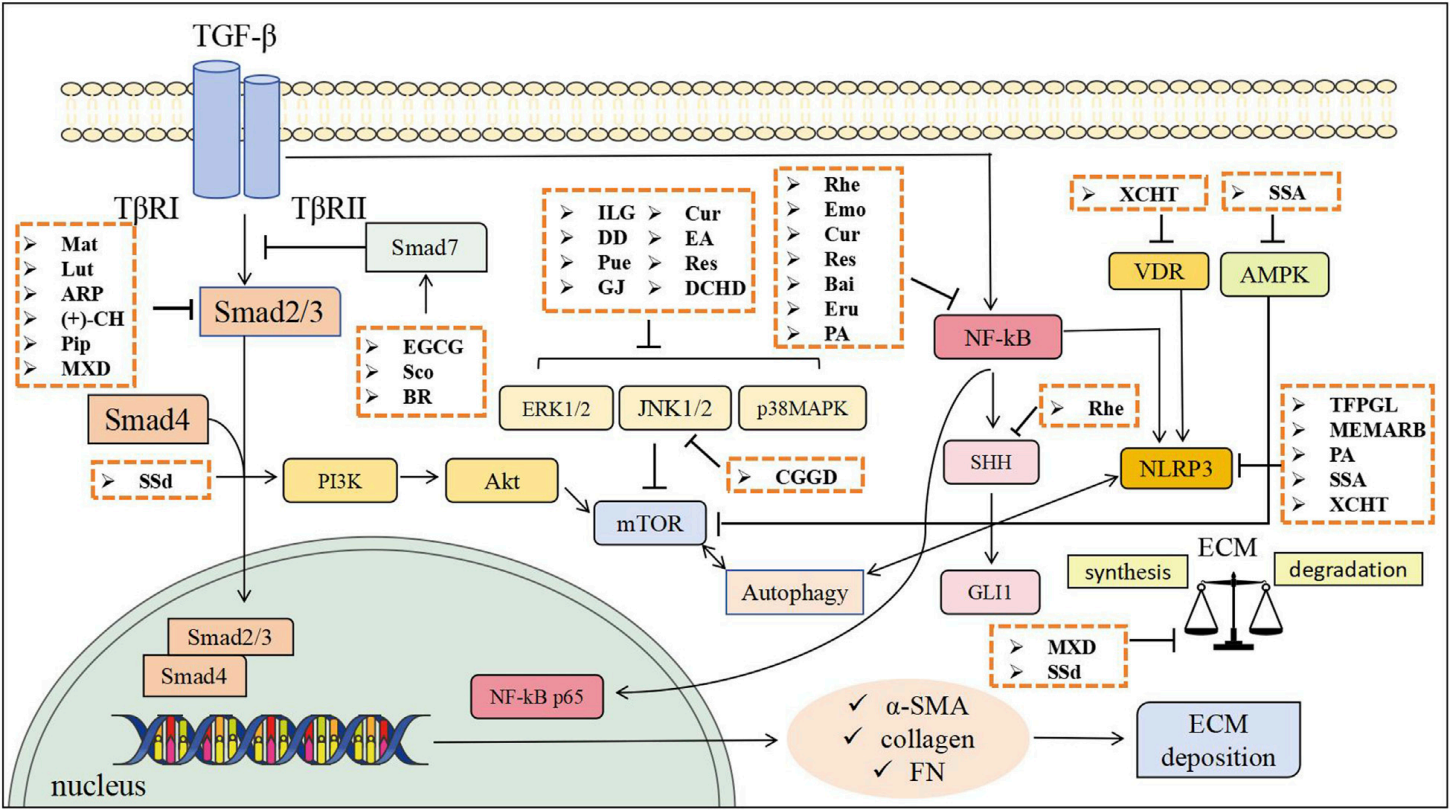
The anti-fibrotic effects of TCM through inhibiting PSCs activation and mitigating ECM accumulation. Mat, Matrine; Lut, Luteolin; ARP, Arecae pericarpium; (+)-CH, Catechin; Pip, Piperine; EGCG, Epigallocatechin gallate; Sco, Scoparone; BR, Berberine; ILG, Isoliquiritigenin; Pue, Puerarin; GJ, Gardenia jasminoides; Cur, Curcumin; EA, Ellagic acid; Res, Resveratrol; Rhe, Rhein; Emo, Emodin; Bai, Baicalin; Eru, Eruberin A; SSA, Saikosaponin A; SSd, Saikosaponin d; TFPGL, Total flavonoids from Psidium guajava leaves; MEMARB, Methanolic extract of Morus alba root bark; PA, Pachymic acid; DD, Dahuang Danshen decoction; CGGD, Chaihu Guizhi Ganjiang Decoction; DCHD, Dachaihu decoction; XCHT, Xiao Chai Hu Tang; MXD, Modified Xiaochaihu Decoction.

**FIGURE 2:**
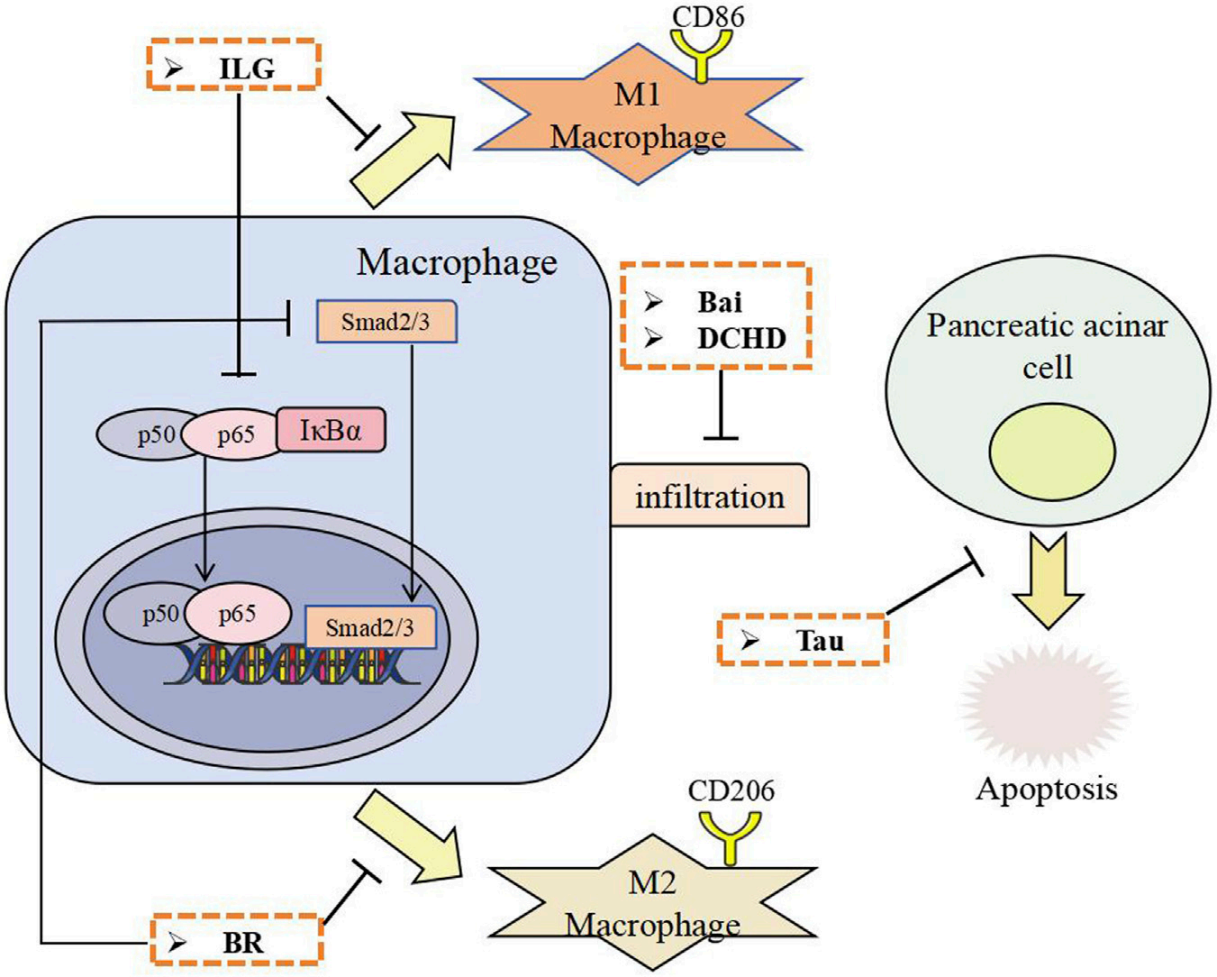
The anti-fibrotic effects of TCM through inhibiting macrophage infiltration and polarization, and inhibiting pancreatic acinar cell apoptosis. ILG, Isoliquiritigenin; BR, Berberine; Bai, Baicalin; Tau, Taurine; DCHD, Dachaihu decoction.

Research suggests that compound formulas and bioactive constituents derived from TCM exhibit multi-target potential against PF. Experimental studies on PF indicate that TCM demonstrate anti-fibrotic properties in preclinical models. Mechanistic studies show these agents may counteract PF by inhibiting PSCs activation, mitigating ECM accumulation, suppressing macrophage infiltration/polarization, and mitigating pancreatic acinar cells apoptosis.PSCs activation involves multiple signaling pathways, including Smad, PI3K/Akt, mitogen-activated protein kinase (MAPK), nuclear factor-κB(NF-κB), Janus tyrosine Kinase (JAK)/Signal Transducer and Activator of Transcription (STAT), and Hedgehog (Hh). Pharmacological evidence indicates that defined TCM compounds primarily modulate TGF-β/Smad, MAPK, NF-κB, and Hh pathways (Jin et al., 2020). Emerging findings suggest targeting NLRP3 inflammasome activation and autophagy modulation represents a potential strategy to inhibit PSC activation and attenuate PF progression. The following sections detail the specific molecular mechanisms by which chemically characterized TCM, including active ingredients, herbal extracts and TCM formulas, exert their anti-fibrotic effects.The schematic diagram of the relevant treatment mechanisms of TCM in PF is shown below (Figures 1, 2).

### 4.1 Inhibition of PSCs activation

#### 4.1.1 TGF-β/Smad signaling pathway

TGF-β is a fundamental signaling protein that modulates numerous physiological processes and is crucial in cell proliferation, differentiation, apoptosis, and ECM synthesis. Its receptors consist of three types: Transforming Growth Factor-β Type I Receptor (TβRI), TβRII, and TβRIII. TGF-β facilitates downstream signaling through binding to certain receptors, which activates various Smad proteins. Smad2 and Smad3 serve crucial functions in signaling as receptor-regulated proteins. Smad7 fulfills its negative regulatory function (Yan et al., 2016). TGF-β is a pivotal modulator of fibrogenesis and significantly contributes to the activation of PSCs and the accumulation of ECM (Biernacka et al., 2011). Preliminary experiments indicate that in PSCs, TGF-β1 enhances the expression of Smad3 while suppressing Smad7 expression; the inhibition of TGF-β signaling by Smad7 mitigates caerulein-induced PF in mice, whereas the absence of a functional Smad7 gene results in exacerbated damage in CP (He et al., 2009; Li et al., 2016; Qian et al., 2010).

Flavonoids: Luteolin (Table 1) is a natural flavonoid found in glycosylated forms in vegetables, botanical drugs, and fruits. It has been reported to have therapeutic effects on a variety of fibrotic diseases (Li et al., 2015; Lv et al., 2025; Ren et al., 2023). Luteolin downregulates TGF-β1 and exerts a beneficial effect on PF in the context of trinitrobenzene sulfonic acid (TNBS)-induced CP (Yu et al., 2018). Catechin, polyphenolic flavonoids found in foods like apples and tea, has shown health benefits and efficacy against diseases, including hepatic fibrosis (Bragança de Moraes et al., 2014). It is reported that catechin hydrate (CH, Table 1) had prophylactic and therapeutic effects on CP and protected against the progression of PF by inactivating the TGF-β/Smad2 signaling pathway (Kweon et al., 2023). Epigallocatechin gallate (EGCG, Table 1), a principal constituent of green tea extract recognized for its antifibrotic activities across various organs, regulates the equilibrium between Smad3 and Smad7 and suppresses TGF-β1-induced activation of PSCs (Meng et al., 2007).

**TABLE 1:** A list of herbal extracts and active ingredients in treating PF.

Drugs	Source (verified in MPNs)	Model	Dose and mode	Type of study	Effects and mechanisms	Target/Pathway	Refs
Luteolin	*Reseda odorata* L	TNBS-induced SD rats; primary rat PSCs	*In vivo*: 12.5,50 mg/kg i.g *In vitro*: 12.5 mM	Both	α-SMA, TGF-β1, IL-1β, IL-6, TNF-α↓	TGF-β/Smad signaling pathway	Yu et al. (2018)
Matrine	*Sophora flavescens* Aiton	TNBS-induced SD rats	100 mg/kg i.p	*In vivo*	α-SMA,TGF-β1, Col-I, Smad2, TβRI, TβRII↓	TGF-β/Smad signaling pathway	Liu et al. (2019a)
Piperine	*Piper nigrum* L	cerulein-induced C57BL/6 mice	*In vivo*: 1, 5, 10 mg/kg i.g *In vitro*: 10, 20, 50 µM	Both	α-SMA, FN 1, Col-I, Col-III, TGF-β/SMAD2/3, TNF-α, IL-1β, IL-6, CCL2, CXCL2↓	TGF-β/Smad signaling pathway	Choi et al. (2019)
Arecae pericarpium water extract	*Areca catechu* L	cerulein-induced C57BL/6 mice; PDGF-BB or TGF-β -induced primary mouse PSCs	*In vivo*: 50, 100, 200 mg/kg i.p *In vitro*: 250 μg/mL	Both	α-SMA, Col-I, FN1, PPP2R2A, Smad2↓	TGF-β/Smad signaling pathway	Kweon et al. (2022)
Catechin hydrate	*Senegalia catechu* (L.f.) P.J.H.Hurter & Mabb.	cerulein-induced C57BL/6 mice; PDGF-BB or TGF-β -induced primary mouse PSCs	*In vivo*: 1, 5 and 10 mg/kg i.p *In vitro*: 150, 200 and 250 µM	Both	α-SMA, FN 1,Col-I, Col-III,COL4A1, p-Smad2/3↓	TGF-β/Smad signaling pathway	Kweon et al. (2023)
Scoparone	*Artemisia capillaris* Thunb	DBTC-induced SD rats; primary rat PSCs	*In vivo*:30, 60 mg/kg, i.g *In vitro*: 0.1,0.2 and 0.4 mM	Both	SOD, E-cadherin, Smad7↑ MDA, α-SMA, Col-I, TGF-β, vimentin, p-Smad2/3↓	TGF-β/Smad signaling pathway	Xu et al. (2016)
Berberine	*Coptis chinensis* Franch	cerulein-induced Swiss albino mice; TGF-β1 -induced RAW 264.7	*In vivo*: 3,10 mg/kg i.p *In vitro*: 3, 10 and 30 μM	Both	p-AMPKα, p-AMPKβ, p-ACC, Smad7, E-cadherin↑ TNF-α, IL-6, IL-1β, TGF-β1, α-SMA, Col-I, Col-III, FN, Snail, Slug, p-Smad2/3, CD206↓	TGF-β/Smad signaling pathway	Bansod et al. (2020)
Epigallocatechin gallate	*Camellia sinensis* (L.) O. Ktze	DDC-induced Wistar rats	50, 100, 200 mg/kg i.g	*In vivo*	Smad7↑ a-SMA, TGF-β1, Smad 3↓	TGF-β/Smad signaling pathway	Meng et al. (2007)
Puerarin	*Pueraria montana var. thomsonii* (Benth.) M.R.Almeida	cerulein-induced C57BL/6 mice; PAF-induced human primary PSCs	*In vivo*: 100 mg/kg i.g *In vitro*: 25, 50, 100 nM	Both	GFAP↑ α-SMA, Col-I, FN, TNF-α, IL-6, JNK 1/2, ERK 1/2, p38 MAPK↓	MAPK signaling pathway	Zeng et al. (2021)
Isoliquiritigenin	*Glycyrrhiza uralensis* Fisch. ex DC.	cerulein-induced C57BL/6 mice; TGF-β -induced human primary PSCs; LPS and IL-4-induced RAW 264.7	*In vivo*: 20, 40 mg/kg i.p *In vitro*: 5, 10 and 20 μM	Both	DUSP5, DUSP10↑ PDGFR, ERK1/2, JNK1/2, FN, CCL2, CCL5, CXCL1, IL-1β, IL-6, TNF-α, IL-4, IL-13, TGF-β1,CD68, F4/80↓	MAPK signaling pathway	Wang et al. (2020)
Gardenia jasminoides extract	*Gardenia jasminoides* J.Ellis	cerulein-induced C57BL/6 mice; PDGF-BB -induced primary mouse PSCs	*In vivo*: 0.1, 1 g/kg i.p *In vitro*: 0.5 mg/mL	Both	α-SMA, Col-I, Col-IV, FN1, p-ERK, p-JNK↓	MAPK signaling pathway	Choi et al. (2020)
Curcumin	*Curcuma longa* L.	PDGF-BB, IL-1β or TNF-α-induced primary mouse PSCs	10, 25 μM	*In vitro*	a-SMA, Col-I, MCP-1, AP-1, p-ERK, p-JNK, p- p38 MAPK↓	MAPK signaling pathway	Masamune et al. (2005a)
		TGF-β-induced LTC-14 PSCs	20 μM	*In vitro*	α-SMA, Col-I, FN1, NF-κBp65↓	NF-κB signaling pathway	Lin et al. (2015)
Rhein	*Rheum palmatum* L	TGF-β-induced LTC-14 PSCs	20 μM	*In vitro*	α-SMA, Col-I, FN1, NF-κBp65↓	NF-κB signaling pathway	Lin et al. (2015)
		cerulein-induced C57BL/6 mice; TGF-β-induced LTC-14 PSCs	*In vivo*: 50 mg/kg i.g *In vitro*: 1, 10, 100 μM	Both	FN1, α-SMA, Col-I, IκB-α↓ SHH/GLI1, NF-κB↓	NF-κB signaling pathway Hedgehog signaling pathway	Tsang et al. (2013)
Resveratrol	*Reynoutria japonica* Houtt	TGF-β-induced LTC-14 PSCs and cerulein-induced AR42J acinar cells	*In vivo*: 20 mg/kg i.g *In vitro*: 50 μM	Both	Akt, p38 MAPK, α-SMA, FN1, TNF-α↓	MAPK signaling pathway	Xia et al. (2018)
		TGF-β-induced LTC-14 PSCs	20 μM	*In vitro*	α-SMA, Col-I, FN1, NF-κBp65↓	NF-κB signaling pathway	Lin et al. (2015)
		TGF-β-induced LTC-14 PSCs	10, 20, 50 μM	*In vitro*	α-SMA, Col-I, FN1, NF-κB, p-AKT	NF-κB signaling pathway	Tsang et al. (2015a)
Ellagic acid	*Rubus idaeus* L	PDGF-BB, IL-1β or TNF-α-induced primary mouse PSCs	10, 25 μM	*In vitro*	a-SMA, Col-I, MCP-1, AP-1, p-ERK, p-JNK, p- p38 MAPK↓	MAPK signaling pathway	Masamune et al. (2005b)
Baicalin	*Scutellaria baicalensis* Georgi	cerulein-induced C57BL/6 mice; TGF-β -induced primary mouse PSCs; primary mouse Bone Marrow-Derived Macrophages	*In vivo*: 100 mg/kg i.p *In vitro*: 50 μg/mL	Both	α-SMA, F4/80, NF-κB, MCP-1, Col-I, TβRI, p-TAK1↓	NF-κB signaling pathway	Fan et al. (2021)
Eruberin A	*Pronephrium penangianum* (Hook.) Holtt	TGF-β-induced LTC-14 PSCs	1, 5, 10, 20 μg/mL	*In vitro*	α-SMA, FN1, SHH, GLI1	Hedgehog signaling pathway	Tsang et al. (2015b)
Pachymic acid	*Poria cocos* (Schw.)Wolf*	cerulein-induced C57BL/6 mice; TGF-β -induced primary mouse PSCs	*In vivo*: 20 mg/kg i.p *In vitro*: 15 μM	Both	α-SMA, Col-I, FN, NF-κB/NLRP3,IL-18, IL-1β↓	NLRP3 inflammasomes	Li et al. (2022)
The extract of total flavonoids from Psidium guajava leaves	*Psidium guajava* L	cerulein-induced C57BL/6 mice	0.372, 0.186 g/kg i.g	*In vivo*	NLRP3, caspase-1, IL-1β, IL-18, Col-I, Col-III, a-SMA↓	NLRP3 inflammasomes	Zhang et al. (2021a)
methanolic extract of Morus alba root bark	*Morus alba* L	cerulein and ethanol-induced Wistar rats	300 mg/kg i.g	*In* *vivo*	GSH/GSSG↑ HSP70, NF-κB, NLRP3, ASC, caspase-1, IL-1β, IL-18↓	NLRP3 inflammasomes	Yuvaraj and Geetha (2018)
Saikosaponin A	*Buplerum chinense* DC.	primary rat PSCs	5, 10 μg/mL	*In vitro*	MMP13↑ α-SMA, FN, Col-I, Col-III, TIMP1, TIMP2, Atg5, Beclin-1, LC3B, NLRP3, Caspase-1, IL-1β, IL-18↓	AMPK/mTOR signaling pathway (Autophagy) NLRP3 inflammasomes	Cui et al. (2020)
Saikosaponin d	*Buplerum chinense* DC.	DBTC-induced Wistar rats; primary rat PSCs	*In vivo*: 2 mg/kg i.g *In vitro*: 5,10 μM	Both	p-PI3K, p-Akt, p-mTOR,MMP2/TIMP2, MMP13/TIMP1,Smad7, P62↑ α-SMA, FN, Col-I, beclin-1, Atg5, LC3-II/LC3-I, TGF-β1, p-Smad3↓	PI3K/Akt/mTOR (Autophagy) TGF-β/Smad signaling pathway	Cui et al. (2019)
Taurine	*Bos taurus domesticus* Gmelin (bile)*	DBTC-induced Wistar rats; DBTC-induced AR42J cells	*In vivo*: 10% taurine diet *In vitro*: 0.8 mM	Both	Bcl-2↑ Bad↓	—	Matsushita et al. (2012)
		DBTC-induced Wistar rats; PDGF-induced primary rat PSCs	*In vivo*: 1% taurine diet *In vitro*: 0.8 mM	Both		—	Shirahige et al. (2008)

↓: downregulation; ↑: upregulation.

* medicines derived from fungi or animals.

Alkaloids: Matrine (Table 1), an alkaloid derived from the roots of *Sophora flavescens* Aiton, has been shown in modern research to possess anti-inflammatory, immunomodulatory, and anti-fibrotic properties (Gao et al., 2012; Zhang et al., 2006). Matrine decreased the expression of TGF-β1, Smad2, and TGF-β receptors (TβRI, TβRII), indicating its inhibitory effect on PF in rats through modulation of TGF-β/Smad signaling (Liu et al., 2019c). Piperine (Table 1), a natural alkaloid derived from black pepper, is known for its anti-inflammatory, antioxidant, and antitumor properties (Srinivasan, 2007). Additionally, studies have demonstrated its protective effects against acute pancreatitis (Bae et al., 2011). Piperine mitigates PF by obstructing TGF-β/Smad 2/3 signaling during CP (Choi et al., 2019). Berberine (BR, Table 1), a natural isoquinoline alkaloid, is extracted from the roots and rhizomes of various medicinal plants, including Berberis species and Coptis chinensis. Reportedly, BR exhibited protective effect on AP and hepatic fibrosis (Choi et al., 2017; Wang et al., 2021; Wang et al., 2024).BR has promising protective effects against cerulein-induced CP by attenuating pancreatic inflammation, PSCs activation and ECM deposition. The protective effects of BR against cerulein-induced CP may be mediated through AMPK-dependent suppression of TGF-β/Smad signaling pathway and inhibition of M2 macrophage polarization (Bansod et al., 2020).

Coumarin: Scoparone (Table 1), the principal bioactive constituent of *Artemisia capillaris* Thunb., is a multifunctional compound with antioxidant and anti-inflammatory properties that exhibits hepatoprotective properties along with various other health benefits (Atmaca et al., 2011; Jang et al., 2006).Scoparone suppressed PSCs activation and PF by inhibiting α-SMA, collagen I, oxidative stress through TGF-β/Smad pathway modulation.

Herbal extracts and TCM formulas: Arecae pericarpium (ARP), a traditional herbal medicine, is used to treat constipation, abdominal distension, and edema (Tey et al., 2021). ARP water extract (Table 1) shown antifibrotic properties in CP by obstructing TGF-β signaling via the inhibition of Smad2 phosphorylation (Kweon et al., 2022). Modified Xiaochaihu Decoction (MXD, Table 2), a Chinese herbal complex prescription, has been used in the treatment of CP for more than 10 years. MXD protected the pancreas from chronic inflammation and fibrosis and enhances exocrine function in a rat CP model induced by DBTC. This likely occured through inhibiting overexpression of TGF-β1, TβRII, and Smad3 in the TGF-β1/Smads pathway (Zhang et al., 2013).

**TABLE 2:** A list of TCM formulas in treating PF.

Drugs	Composition (verified in MPNs)	Model	Dose and mode	Type of study	Effects and mechanisms	Target pathway	Refs
Modified Xiaochaihu Decoction	*B*upleurum falcatum L., *Scutellaria baicalensis* Georgi, *Pinellia ternata* (Thunb.) Makino, *Glycyrrhiza uralensis* Fisch. ex DC., *Prunus persica* (L.) Batsch	DBTC-induced Wistar rats	10 g/kg i.g	*In vivo*	TGF-β1, TβRII, Smad3↓	TGF-β/Smad signaling pathway	Zhang et al. (2013)
		DBTC-induced Wistar rats	10 g/kg i.g	*In vivo*	MMP13, TIMP1↑ Col-I, Col-III↓	—	Zhang et al. (2017a)
Dahuang Danshen decoction	*Rheum palmatum* L.*,* *Salvia miltiorrhiza* Bunge	DDC-induced SD rats	1.37, 2.74, 5.48 g/kg i.g	*In vivo*	GSH, SOD, Nrf2, NQO1, GPX1, HO-1↑ α-SMA, Col-I, Col-III, TNF-α, IL-6, ROS, Keap-1, GRP, JNK, MMK-3/p38↓	MAPK signaling pathway	Liang et al. (2021)
Dachaihu decoction	*Bupleurum falcatum* L.*, Scutellaria baicalensis* Georgi, *Citrus × aurantium *L.*, Paeonia lactiflora* Pall., *Pinellia ternata* (Thunb.) Makino*, Rheum palmatum* L., *Zingiber officinale* Roscoe*, Ziziphus jujuba* Mill	cerulein-induced C57BL/6 mice	5.5, 11, 22 g/kg i.g	*In vivo*	α-SMA, Col-I,f IL-6, MCP-1, TNF-α, p-JNK, p-ERK, p-P38, JNK1, ERK1, P38↓	MAPK signaling pathway	Li et al. (2025)
		L-arginine-induced KunMing mice	14 g/kg i.g	*In vivo*	IL-6, MCP-1, MIP-1α, FN↓	—	Duan et al. (2017)
Xiao Chai Hu Tang	*Bupleurum falcatum* L., *Scutellaria baicalensis* Georgi, *Pinellia ternata* Makino, *Zingiber officinale* Roscoe, *Panax ginseng* C.A.Mey., *Ziziphus jujuba* Mill., *Glycyrrhiza uralensis* Fisch. ex DC.	cerulein-induced C57BL/6 mice	15, 30, 60 g/kg i.g	*In vivo*	VD3, VDR↑ Col-I, Col-III, α-SMA, NLRP3, IL-1β, TNF-α, IL-6↓	NLRP3 inflammasomes	Zhang et al. (2023)
Chaihu Guizhi Ganjiang Decoction	*Bupleurum chinense* DC.*, Neolitsea cassia* (L.) Kosterm.*, Zingiber officinale* Roscoe*, Trichosanthes kirilowii* Maxim., *Scutellaria baicalensis* Georgi, *Glycyrrhiza uralensis* Fisch. ex DC., *Crassostrea gigas* (Thunberg)*	DBTC-induced SD rats; primary rat PSCs	*In vivo*: 1.44 g/kg i.g *In vitro*: 20% or 50% serum CGGD	Both	JNK/mTOR↑ α-SMA, COLI, FN, MMP2,TIMP2, Atg5, Beclin-1,LC3B↓	JNK/mTOR signaling pathway (Autophagy)	Cui et al. (2021)

* medicines derived from animals.

A series of active ingredients and TCM formulas demonstrate significant potential in alleviating PF primarily by modulating the TGF-β/Smad signaling pathway. Flavonoids like luteolin, catechin, and EGCG, alkaloids including matrine, piperine, and BR, the coumarin scoparone and herbal extracts such as ARP water extract and MXD all converge on inhibiting this core fibrogenic pathway, thereby reducing activation of PSCs, ECM deposition, and key markers like α-SMA and collagen. Notably, compounds like berberine exhibit additional mechanisms involving AMPK activation and immunomodulation (e.g., suppressing M2 macrophage polarization).

#### 4.1.2 MAPK signaling pathway

The MAPK pathway is a crucial signaling path in eukaryotic cells. In reaction to external stimuli, MAPKs influence various cellular processes, including proliferation, apoptosis, and survival, and can enhance the expression of inflammatory cytokines in the pancreas (Pearson et al., 2001). Three extensively researched MAPK signaling pathways include Extracellular regulated protein kinases (ERK)1/2, c-Jun N-terminal kinase (JNK), and p38 MAPK. In a mouse model of CP, there is an elevation of ERK, JNK, and p38 MAPK, with PSCs identified as the source of MAPK production (Xu et al., 2018). The canonical signaling cascade for ERK1/2 is Ras-Raf-MEK-ERK. The ERK pathway influences the migration, activation, and matrix formation of PSCs (Schwer et al., 2012). Mitogen-activated protein (MEK) inhibitors mitigate pancreatic inflammatory damage and fibrosis caused by caerulein, thereby affirming the significant regulatory function of the MAPK signaling pathway in PF (Halbrook et al., 2017).

Flavonoids: Puerarin (Table 1), the principal bioactive flavonoid compound derived from the traditional Chinese herb Radix Puerariae. Accumulating evidence from recent studies has demonstrated its therapeutic efficacy in attenuating fibrotic progression across multiple organ systems, including hepatic, pulmonary, renal, and cardiac fibrosis (Li et al., 2020; Zhang et al., 2019; Zhou et al., 2017). Puerarin markedly suppressed the phosphorylation of MAPK family proteins (JNK1/2, ERK1/2, and p38 MAPK) in PSCs in a dose-dependent manner for the treatment of CP (Zeng et al., 2021). Isoliquiritigenin (Table 1), a bioactive chalcone-type flavonoid derived from *Glycyrrhiza uralensis* Fisch., possesses diverse pharmacological properties, including antioxidant, anti-inflammatory, and hepatoprotective activities (Jin et al., 2016; Na et al., 2018). Isoliquiritigenin exerted therapeutic effects against AP by suppressing oxidative stress (Liu et al., 2018). Isoliquiritigenin notably alleviated PF and infiltration of macrophages in a model of caerulein-induced murine CP. *In vitro* investigations using human PSCs demonstrated that Isoliquiritigenin significantly suppressed both proliferation and activation of human PSCs, potentially through its inhibitory effects on ERK1/2 and JNK1/2 signaling pathways (Wang et al., 2020).

Polyphenols: Resveratrol (Table 1), is a natural polyphenolic constituent found in grapes, berries, knotweed and many other food products, and has been widely reported to have antioxidant and antitumor properties (Burns et al., 2002). The beneficial effects of trans-resveratrol are supported by extensive research, particularly *in vitro* studies (Lagouge et al., 2006). Trans-resveratrol suppressed PSCs activation, reducing fibrogenesis severity, and alleviated acinar injury by downregulating Akt and p38 MAPK pathways and attenuating RORγt activity (Xia et al., 2018). Curcumin (Table 1), derived from *Curcuma longa* Linn (turmeric), exhibits anti-inflammatory, antioxidant, and antifibrotic properties. It protects against AP in rats, bleomycin-induced pulmonary fibrosis in mice, and carbon tetrachloride-induced liver fibrosis (Gukovsky et al., 2003; Kang et al., 2002; Punithavathi et al., 2000). Curcumin effectively suppressed IL-1β- and TNF-α-induced activation of MAPK signaling pathways, including ERK, JNK, and p38 MAPK, thereby inhibiting the activation of PSCs (Masamune et al., 2005a). Ellagic acid (Table 1) is a plant-derived polyphenol found in fruits and nuts such as raspberries, strawberries, walnuts, grapes, and black currants (Priyadarsini et al., 2002), and has been shown in pharmacological studies to have a protective effect against a variety of fibrotic diseases (Chen J et al., 2023; Li et al., 2021; Mannino et al., 2023). Ellagic acid inhibits pancreatic stellate cell activation by inhibiting all three classes of MAP kinases (Masamune et al., 2005b).

Herbal extracts and TCM formulas: Gardenia jasminoides (GJ, Table 1), an evergreen flowering plant belonging to the Rubiaceae family, has a longstanding history of application in China, widely utilized as a traditional herbal remedy for managing inflammatory conditions and fever (Hou et al., 2024). Previous research has substantiated the anti-inflammatory efficacy of GJ in the context of AP (Jung et al., 2008). The study showed that GJ extract could attenuate the severity of CP and PF by inhibiting ERK and JNK activation during CP (Choi et al., 2020). Dahuang Danshen decoction (DD, Table 2) consists of *Rheum palmatum* L. stem and *Salvia miltiorrhiza* Bge. It has the effect of activating blood circulation and removing blood stasis. Studies have demonstrated the protective effect of *Rheum palmatum L. stem* and *Salvia miltiorrhiza Bge*. against acute pancreatitis (Feng et al., 2024). DD reduces diethyldithiocarbamate (DDC)-induced CP fibrosis by modulating inflammatory mediators, relieving oxidative and ER stress, and inhibiting PSCs activation via suppressing JNK and MKK3/p38 pathways (Liang et al., 2021). Dachaihu decoction (DCHD, Table 2) is a traditional Chinese medicine formula that comes from the classic medical book Shang Han Lun written by Zhonging Zhang of the Eastern Han Dynasty. DCHD has been widely used in the clinical treatment of AP. Recently, it was used to treat patients with CP, with studies showing it effectively alleviates CP symptoms. DCHD might alleviate pancreatic inflammatory cell infiltration and fibrosis by regulating the MAPK signaling pathway (Li et al., 2025).

Numerous active ingredients and TCM formulas demonstrate potent anti-fibrotic effects in CP primarily through suppression of the MAPK signaling pathway. Flavonoids such as puerarin and isoliquiritigenin, polyphenols including resveratrol, curcumin, and ellagic acid, extracts from GJ, as well as TCM formulas like DD and DCHD all converge on inhibiting key MAPK components (ERK, JNK, and p38), thereby attenuating PSCs activation, reducing inflammation, oxidative stress, and ECM deposition. This collective evidence highlights a shared mechanistic theme across diverse natural products: targeting MAPK cascades to disrupt fibrotic signaling networks.

#### 4.1.3 NF-κB signaling pathway

The NF-κB family of transcription factors serves as crucial regulators of immunological development, immune response, inflammation, and cancer. It has five protein monomers: p65/RelA, RelB, c-Rel/Rel, p50/NF-κB1, and p52/NF-κB2. The connections among NF-κB dimers, the inhibitor of NF-κB (IκB), and the IκB kinase (IKK) complexes form the NF-κB signaling pathway (Mitchell et al., 2016). Fundamental research has demonstrated that the activation of the NF-κB pathway directly exacerbates the severity of CP and contributes to heightened fibrosis (Huang et al., 2013). Nonetheless, it has been proposed that the function of NF-κB is complex across many stages of pancreatitis, with outcomes contingent upon the diverse experimental models and methodologies employed by different research teams (Neesse and Ellenrieder, 2017).

Polyphenols: Four phenolic compounds, rhein, emodin, curcumin, and resveratrol (Table 1), reduced the expression levels of Acta2, Col-I, FN, as well as the nuclear expression of NF-κB in the rat PSCs line LTC-14 following TGF-β stimulation (Lin et al., 2015). This indicates that their mechanism of action against PF primarily involves inhibiting the activation of the NF-κB signaling pathway. However, this effect is limited to *in vitro* studies and only impacts the NF-κB target. Another study found that resveratrol’s antifibrotic mechanism was related to the inhibition of NF-κB activation and the reduction of protein kinase B (Akt) phosphorylation (Tsang et al., 2015a).

Flavonoids: Further research is needed to clarify the mechanisms of these compounds. Baicalin (Table 1), the primary bioactive component of *Scutellaria baicalensis* Georgi. It exhibits a broad spectrum of pharmacological activities, including anti-inflammatory and anti-fibrotic effects on organs such as the liver, kidneys, and lungs (Dinda et al., 2017; Wang et al., 2015; Zaghloul et al., 2022; Zhao et al., 2020). One finding indicated that baicalin could impede the activation of PSCs by down-regulating the TGF-β1/TGF -βR1/TAK1/NF-κB signaling pathway, hence mitigating PF (Fan et al., 2021).

Others compounds: Eruberin A (Table 1) can be extracted from *Pronephrium penangianum* (Hook.) Holtt. This plant has a long history of use as a folk medicine in Chinese traditional medicine. Eruberin A markedly inhibited NF-κB activation and PI3K/Akt phosphorylation, reducing fibrotic mediator expression in PSCs. Its antifibrotic action was tied to suppressing the PI3K/Akt/NF-κB signaling pathway (Tsang et al., 2015b).

Active ingredients exhibit anti- PF effects predominantly through inhibition of the NF-κB pathway, though with varying mechanistic breadth. Polyphenols, including rhein, emodin, curcumin, and resveratrol, consistently suppress PSCs activation and reduce fibrotic marker expression by blocking NF-κB nuclear translocation, although evidence remains largely limited to *in vitro* models. Flavonoids such as baicalin target a broader upstream spectrum, inhibiting the TGF-β1/TβR1/TAK1/NF-κB axis. Other structurally distinct compounds like eruberin A, and to some extent resveratrol, demonstrate enhanced efficacy by concurrently modulating complementary pathways such as PI3K/Akt alongside NF-κB. Inhibition of NF-κB is a widely recognized therapeutic strategy for PF.

#### 4.1.4 Hedgehog signaling pathway

The Hh signaling pathway comprises three ligands, including Sonic hedgehog (Shh), Indian hedgehog (Ihh), and Desert hedgehog (Dhh), two transmembrane receptor proteins (Ptch1 and Ptch2), one signal transduction factor (Smo), and three transcription factors, including Glioma-Associated Oncogene (Gli)1, Gli2, and Gli3. Activated PSCs were seen to express Ptch1 and Smo (Shinozaki et al., 2008). Ihh augmented the migration of PSCs and elevated matrix metalloproteinase (MMP)1 expression.Ihh prompted the increase of Gli1 in the nucleus of PSCs, indicating that Ihh may activate the Gli1-dependent signaling pathway. Paracrine Hh signaling has been shown to activate and promote the proliferation of myofibroblasts in pancreatic tissues, as well as induce the formation of MMP (Bai et al., 2016). Consequently, Hh signaling is a crucial mechanism for PSCs activation and ECM synthesis during CP fibrosis. Inhibition of Hh signaling after application of vismodegib, a Hh pathway inhibitor, ameliorates L-arginine or caerulein-induced CP severity (Iyer et al., 2024).

Rhein (Table 1), a natural anthraquinone derivative extracted from *Rheum palmatum* L., is a yellow crystalline compound. It has been used as a mild laxative and astringent in Chinese traditional medicine since ancient times. Rhein reduced α-SMA, FN1, Col-I, and Shh expression in a caerulein-induced CP mouse model, alleviating PF by inhibiting the Shh/Gli1 pathway. Shh and Gli1 expression levels in pancreatic tissue were positively correlated with PF severity, highlighting the Shh/Gli1 pathway’s key role in PF development (Tsang et al., 2013).

#### 4.1.5 NLRP3 inflammasomes

NLRP3 inflammasomes comprise a soluble pattern recognition receptor connected to NLRP3 protein by the amino-terminal pyridine structural domain, which links to the N-terminal end of apoptosis-associated speck-like protein, while the C-terminal end of ASC associates with procaspase-1; collectively, these components constitute NLRP3 inflammasomes (Zhang et al., 2021a). This is a recently discovered cytoplasmic signaling complex that facilitates the activation of powerful inflammatory mediators and is especially pertinent to metabolic disorders, multiple sclerosis, inflammatory bowel disease, and autoimmune and autoinflammatory conditions (Mangan et al., 2018). NLRP3 participates in pancreatic inflammation and the synthesis of proinflammatory cytokines. Inhibition of NLRP3 activation suppresses PSCs activation, thereby postponing the fibrotic process in CP (Zhang G. X. et al., 2017). Furthermore, it has been shown that inhibition of the NF-κB pathway diminishes NLRP3 expression, therefore alleviating the severity of CP (Kanak et al., 2017).

Active ingredients: Saikosaponin A (SSa, Table 1) is one of the main active ingredients of *Buplerum chinense* DC., which possesses a variety of pharmacological activities including anti-inflammation (Piao et al., 2020). SSa inhibits autophagy in PSCs and suppresses NLRP3, indicating a link between autophagy and NLRP3 during the suppression of PSCs (Cui et al., 2020). Pachymic acid (PA, Table 1), a triterpenoid derived from Poria cocos, exhibits anti-inflammatory and anticancer properties (Cheng et al., 2015). PA repressed cerulein or TGF-β-induced activation of NF- κB/NLRP3 inflammasome activation to mitigate PSCs activation and PF (Li et al., 2022).

Herbal extracts and TCM formulas: Psidium guajava is a well-known traditional medicinal plant widely used in folk medicine. Animal studies have shown that extracts of guava leaves can inhibiting effects on chronic inflammation (Jayachandran et al., 2018; Luo et al., 2019). The extract of total flavonoids from Psidium guajava leaves (TFPGL, Table 1) markedly diminished the production of caspase-1, IL-1β, and IL-18, indicating that it mitigates pancreatic inflammation and fibrosis via inhibiting NLRP3 activation (Zhang et al., 2021b). *Morus alba* L., or white mulberry, native to northern China, has significant ethno medicinal value. Its root bark contains bioactive compounds with antibacterial, antiviral, and antioxidant properties (Chan et al., 2016). The methanolic extract of Morus alba root bark (MEMARB, Table 1) may prevent CP via influencing heat shock protein 70 (HSP70) in relation to NF-κB and NLRP3 activation (Yuvaraj and Geetha, 2018). Xiao Chai Hu Tang (XCHT, Table 2) also comes from Shang Han Lun. In Shang Han Lun, XCHT addressed symptoms like poor appetite, nausea, vomiting, and upper abdominal pain, akin to CP symptoms (Zhang et al., 2023). It reported that XCHT suppressed PF and chronic inflammation in a caerulein-induced CP model by augmenting VD3/VDR expression and reducing the release of NLRP3-associated inflammatory mediators.

Multiple active ingredients and TCM formulas exert anti-PF effects primarily by inhibiting the NLRP3 inflammasome pathway. TFPGL and SSa suppresses NLRP3 activation, while SSa additionally inhibits autophagy. PA and MEMARB target both NF-κB/NLRP3 pathways. XCHT enhances VD3/VDR expression to suppresses NLRP3 activation. These findings underscore NLRP3 inflammasome inhibition as a common mechanistic target across diverse compounds, though further *in vivo* and clinical studies are warranted to confirm efficacy and explore additional mechanisms.

#### 4.1.6 Autophagy and PF

Autophagy is a conserved catabolic mechanism that sequesters cytoplasmic constituents within a double-membrane vesicle known as an autophagosome, which is then transported to lysosomes for breakdown and recycling (Eskelinen, 2019). Autophagy is a dynamic process governed by a collection of proteins expressed by autophagy-related genes (Atg). Previous work has established that autophagy plays a role in the activation of PSCs, and that the inhibition of autophagy in PSCs concurrently suppresses their activation (Li et al., 2018). It is postulated that PSCs may destroy cytoplasmic lipid droplets via autophagy to supply raw materials and energy for quiescent PSCs, hence facilitating their activation (Sousa et al., 2016). The mammalian target of rapamycin (mTOR) is crucial for controlling protein synthesis, cell cycle distribution, cell proliferation, and apoptosis, and serves as a central hub for modulating cellular autophagy activity. Inhibition of mTOR promotes autophagy, whereas activation of mTOR suppresses the beginning of autophagy (Jung et al., 2009).

Active ingredients: Saikosaponin d (SSd, Table 1), another active component of *Buplerum chinense* DC., prevented PF by inhibiting PSCs autophagy via the PI3K/Akt/mTOR pathway, which interacted with the TGF-β1/Smads pathway (Cui et al., 2019). SSA inhibited PSCs activation by inhibiting PSCs autophagy and the NLRP3 inflammasome via the Adenosine 5′-monophosphate-activated protein kinase (AMPK)/mTOR pathway.

Herbal extracts and TCM formulas: Chaihu Guizhi Ganjiang Decoction (CGGD, Table 2) is a TCM formula that was first described by Zhongjing Zhang in “Shang Han Lun”. It has been widely used in the clinical treatment of digestive in TCM. CGGD suppressed autophagy by down-regulating Atg5, Beclin-1, and LC3B, while enhancing the phosphorylation of mTOR and JNK in pancreatic tissues and PSCs. CGGD mitigated PF and the activation of PSCs by suppressing PSCs autophagy via the JNK/mTOR signaling pathway (Cui et al., 2021).

Together, these results emphasize the importance of mTOR mediated inhibition of PSCs autophagy as a shared antifibrotic mechanism among active ingredients and complex TCM formulas.

### 4.2 Mitigating ECM accumulation

In pancreatic injury, PSCs are stimulated to generate substantial quantities of ECM for tissue repair and regeneration at sites of fibrogenesis; however, an imbalance between ECM synthesis and degradation may induce fibrosis of the pancreatic parenchyma, ultimately causing irreversible morphological damage to the organ. Consequently, equilibrating the synthesis and breakdown of the ECM is regarded as an effective approach for addressing PF (Schneider et al., 2001). The degradation of ECM is primarily governed by the regulation of degradative enzyme systems within the organism. Among these, MMPs are capable of selectively degrading various ECM components. The activity and expression levels of MMPs are subsequently inhibited by tissue inhibitors of metalloproteinases (TIMPs). Therefore, modulating the expression of MMPs or TIMPs, as well as regulating the activity of MMPs/TIMPs-related signaling pathways, can influence ECM degradation.

Certain active ingredients and TCM formulas address PF by restoring the critical balance between ECM synthesis and degradation, primarily by modulating the MMP/TIMP system. The triterpenoid saponin SSd enhances ECM breakdown by increasing the ratio of MMPs to TIMPs (Cui et al., 2019). Similarly, the TCM formula MXD promotes collagen degradation specifically by upregulating the expression of MMP13 (Zhang et al., 2017b). These interventions highlight a distinct therapeutic strategy focused not on suppressing PSCs activation directly, but on facilitating the clearance of accumulated fibrotic tissue, thereby contributing to the resolution of fibrosis and restoration of pancreatic architecture.

### 4.3 Inhibiting macrophage infiltration and polarization and pancreatic acinar cell apoptosis

While PSCs activation is a central focus in PF research, emerging evidence highlights the critical roles of macrophages and acinar cells in its pathogenesis. Macrophages contribute to PF by secreting TGF-β to activate PSCs and by sustaining a pro-inflammatory microenvironment (Michalski et al., 2007; Schmid-Kotsas et al., 1999). Beyond immune cells, acinar cell apoptosis-releasing DAMPs like high mobility group box 1 (HMGB1) exacerbates pancreatic injury (Zhan et al., 2019).

The alkaloid berberine inhibits both TGF-β1/Smad signaling and M2 macrophage polarization via AMPK (Bansod et al., 2020); the flavonoid baicalin reduces macrophage recruitment by blocking MCP-1 release from PSCs (Fan et al., 2021); and another flavonoid, isoliquiritigenin suppresses M1 macrophage polarization by inhibiting NF-κB (Wang et al., 2020). The TCM formula DCHD also attenuates PF by suppressing macrophage infiltration (Duan et al., 2017). The amino acid derivative taurine, extracted from ox bile, is a TCM with over two millennia of use in treating fever, inflammation, and gallbladder issues. Taurine suppressed apoptosis of pancreatic acinar cells and mitigated PF in experimental CP (Matsushita et al., 2012). These findings underscore a multi-cellular therapeutic strategy, where active ingredients concurrently target PSCs, macrophage-driven inflammation to disrupt the progression of PF.

The pathogenesis of PF is orchestrated by intricate cross-talk among acinar cells, macrophages PSCs, which converge on shared signaling pathways. Notably, TCM interventions target this multicellular crosstalk through a system-level approach. Active ingredients from TCM, such as berberine and isoliquiritigenin simultaneously modulate multiple nodes within this network: they suppress macrophage M1 or M2 polarization, and inhibit PSCs activation, primarily through downstream regulation of converging pathways TGF-β/Smad or MAPK. By targeting multiple cell types and pathways synergistically, TCM offers a holistic strategy to disrupt the vicious cycle of pancreatic fibrogenesis, representing a unique therapeutic advantage over conventional single-target agents.

## 5 Comparison with modern anti-fibrotic therapies

Pirfenidone and nintedanib are established as first-line therapies for idiopathic pulmonary fibrosis and progressive pulmonary fibrosis (Raghu et al., 2022). However, clinical studies have reported that these agents are associated with considerable adverse effects, including photosensitive rash and gastrointestinal disturbances, which can substantially impair patient quality of life, contribute to economic burden, and often lead to treatment discontinuation (Spagnolo et al., 2021). In contrast, TCM offers a multi-targeted therapeutic strategy with emerging potential in the management of fibrotic diseases. For instance, Shengxian Decoction, a classic formula documented in authoritative Chinese materia medica, has demonstrated efficacy comparable to pirfenidone in key metrics of pulmonary fibrosis at a medium dose (78 mg/kg/d), suggesting its promise as an alternative treatment (Liang et al., 2024). Similarly, *Elephantopus scaber* L., an herb used in TCM for heat-clearing and detoxification, mitigated bleomycin-induced pulmonary inflammation and fibrosis *in vivo* by attenuating neutrophil infiltration and reducing fibroblast foci, showing effectiveness similar to pirfenidone (Jia et al., 2024). These examples underscore the potential of TCM in treating fibrotic conditions. In the specific context of PF, although pirfenidone and nintedanib have been validated in pulmonary settings, their efficacy remains uncertain for pancreatic applications, owing to a narrow focus on isolated pathways such as growth factor inhibition. Conversely, TCM employs a holistic approach that simultaneously addresses multiple pathological processes, including chronic inflammation, oxidative stress, PSCs activation, and immune dysregulation. This broad mechanistic engagement may not only inhibit fibrosis but also promote tissue repair. Nevertheless, TCM faces significant challenges, such as lack of standardization, undefined pharmacokinetics, and insufficient evidence from large-scale randomized controlled trials targeting PF. Therefore, while TCM represents a promising complementary strategy with multi-modal mechanisms, its clinical translation requires further rigorous validation to achieve the reproducibility and regulatory approval accorded to conventional antifibrotic agents.

## 6 Critical appraisal and future directions

First, although numerous active ingredients demonstrate efficacy in modulating key pathways such as TGF-β/Smad, MAPK, and NF-κB, the molecular mechanisms underlying their actions remain incompletely elucidated. For many agents, including multi-target compounds like berberine and baicalin, direct molecular targets, binding affinities, and precise pharmacodynamic relationships are still poorly defined. To establish causal mechanistic links, future studies should incorporate advanced approaches such as chemical proteomics, target deconvolution strategies, and genetic perturbation models (e.g., CRISPR/Cas9 or RNAi). Clarifying these mechanisms is essential for distinguishing primary targets from downstream effects and for rational optimization of lead compounds. Moreover, while the discussed phytochemicals (such as berberine, baicalin, and curcumin) are widely used within the TCM system, it must also be acknowledged that they are widely present in nature.

Second, a major obstacle to clinical translation is the frequent disconnect between pharmacokinetic properties and pharmacodynamic effects. Promising *in vitro* activities of constituents such as resveratrol and curcumin are often compromised by poor bioavailability (e.g., curcumin’s <1% oral bioavailability), rapid metabolism, and insufficient accumulation in pancreatic tissue (Wang et al., 2022). Effective concentrations in cell-based assays commonly exceed physiologically achievable plasma levels. To bridge this Pharmacokinetic-Pharmacodynamic gap, the development of advanced drug delivery systems, such as nanoparticles, liposomes, or phytosomes engineered for pancreatic targeting, is critical to enhance biodistribution and achieve therapeutically relevant exposure profiles.

Third, heavy reliance on rodent models induced by caerulein or DBTC constitutes another significant limitation. These models produce acute pancreatic injury and accelerated fibrosis that may not fully recapitulate the chronic, low-grade inflammatory and metabolic dysregulation characteristic of human CP. Moreover, conventional monocultures of PSCs fail to capture the multicellular complexity of the pancreatic microenvironment. There is a compelling need to adopt more physiologically relevant models, such as patient-derived organoids, 3D heterotypic spheroids incorporating PSCs, acinar cells, and macrophages, and genetically engineered mouse models that better mimic human disease progression.

Finally, the translational gap between encouraging preclinical results and demonstrated clinical efficacy. A notable scarcity of high-quality clinical trials investigating TCM for PF persists; existing studies are predominantly published in Chinese and are typically limited by small sample sizes (Durgaprasad et al., 2005). Furthermore, no interventions based on TCM have advanced to clinical trials approved by the U.S. Food and Drug Administration (FDA) for CP or PF. Barriers to clinical advancement include insufficient characterization of TCM formulas, a lack of pharmacokinetic data for active ingredients, undefined dosing regimens, and incomplete safety profiles. Future research must prioritize extract standardization, innovative formulation design to improve bioavailability and targeting, and the execution of rigorously controlled, biomarker-driven early-phase clinical trials.

## 7 Conclusion

Currently, no single pharmacological agent has received approval from the FDA to reverse PF. TCM offers a unique and valuable approach to combating PF, characterized by their multi-target mechanisms, ability to modulate complex pathological networks, and favorable safety profiles. These achievements highlight the potential of TCM as a source of novel therapeutic candidates for PF. However, to fully translate these promising findings from bench to bedside, the field necessitates a paradigm shift from phenomenological observation to mechanism-driven, pharmacokinetically-aware, and clinically-relevant pharmacological research. By addressing these critical gaps, it will establish an essential scientific foundation for optimizing clinical strategies in PF prevention and treatment.

## Glossary

ADM: Acinar-to-Ductal Metaplasia
Akt: Protein kinase B
AMPK: Adenosine 5′-monophosphate-activated protein kinase
AP: Acute pancreatitis
Atg: Autophagy-related genes
CK: Cytokeratin
Col-I: Collagen type I
Col-III: Collagen type III
CP: Chronic pancreatitis
DAMP: Damage-associated molecular pattern
DDC: Diethyldithiocarbamate
Dhh: Desert hedgehog
ECM: Extracellular matrix
ERCP: Endoscopic retrograde cholangiopancreatography
ERK: Extracellular regulated protein kinases
ESWL: Extracorporeal shock wave lithotripsy
FDA: U.S. Food and Drug Administration
Gli: Glioma-Associated Oncogene
HA: Hyaluronic acid
Hh: Hedgehog
HMGB1: High mobility group box 1
HSP70: Heat shock protein 70
Ihh: Indian hedgehog
IKK: Inhibitor of nuclear factor kappa-B kinase
IL: Interleukin
IκB: Inhibitor of Nuclear factor-κB
JAK: Janus tyrosine Kinase
JNK: c-Jun N-terminal kinase
MAPK: Mitogen-activated protein kinase pathway
MCP-1: monocyte chemotactic protein 1
MEK: Mitogen-activated protein
MMP: Matrix metalloproteinase
mTOR: Mammalian target of rapamycin
NF-κB: Nuclear factor-κB
NLRP3: Nod-like receptor family pyrin domain containing 3
PDGF: Platelet-derived growth factor
PF: pancreatic fibrosis
PI3K: Phosphoinositide 3-kinase
PSCs: Pancreatic stellate cells
Shh: Sonic hedgehog
STAT: Signal Transducer and Activator of Transcription
TCM: Traditional Chinese medicine
TGF-β: Transforming Growth Factor-β
TIMPs: Tissue inhibitors of metalloproteinases
TNBS: Trinitrobenzene sulfonic acid
TNF-α: Tumor necrosis factor-α
TβR: Transforming Growth Factor-β receptor
α-SMA: α-smooth muscle actin
